# Respiratory syncytial virus nonstructural proteins 1 and 2: Exceptional disrupters of innate immune responses

**DOI:** 10.1371/journal.ppat.1007984

**Published:** 2019-10-17

**Authors:** Koen Sedeyn, Bert Schepens, Xavier Saelens

**Affiliations:** 1 VIB-UGent Center for Medical Biotechnology, Ghent, Belgium; 2 Department for Biomedical Molecular Biology, Ghent University, Ghent, Belgium; 3 Department of Biochemistry and Microbiology, Ghent University, Ghent, Belgium; University of Alberta, CANADA

## Abstract

Human respiratory syncytial virus (RSV) is the most important cause of acute lower respiratory tract disease in infants worldwide. As a first line of defense against respiratory infections, innate immune responses, including the production of type I and III interferons (IFNs), play an important role. Upon infection with RSV, multiple pattern recognition receptors (PRRs) can recognize RSV-derived pathogen-associated molecular patterns (PAMPs) and mount innate immune responses. Retinoic-acid-inducible gene-I (RIG-I) and nucleotide-binding oligomerization domain-containing protein 2 (NOD2) have been identified as important innate receptors to mount type I IFNs during RSV infection. However, type I IFN levels remain surprisingly low during RSV infection despite strong viral replication. The poor induction of type I IFNs can be attributed to the cooperative activity of 2 unique, nonstructural (NS) proteins of RSV, i.e., NS1 and NS2. These viral proteins have been shown to suppress both the production and signaling of type I and III IFNs by counteracting a plethora of key host innate signaling proteins. Moreover, increasing numbers of IFN-stimulated genes (ISGs) are being identified as targets of the NS proteins in recent years, highlighting an underexplored protein family in the identification of NS target proteins. To understand the diverse effector functions of NS1 and NS2, Goswami and colleagues proposed the hypothesis of the NS degradasome (NSD) complex, a multiprotein complex made up of, at least, NS1 and NS2. Furthermore, the crystal structure of NS1 was resolved recently and, remarkably, identified NS1 as a structural paralogue of the RSV matrix protein. Unfortunately, no structural data on NS2 have been published so far. In this review, we briefly describe the PRRs that mount innate immune responses upon RSV infection and provide an overview of the various effector functions of NS1 and NS2. Furthermore, we discuss the ubiquitination effector functions of NS1 and NS2, which are in line with the hypothesis that the NSD shares features with the canonical 26S proteasome.

## Introduction

Human respiratory syncytial virus (RSV) is a negative strand RNA virus belonging to the family Pneumoviridae. RSV is a major cause of acute lower respiratory tract infections in the pediatric population and is increasingly recognized as an important cause of severe respiratory disease in the elderly [1–3]. Despite the major clinical impact of RSV on human health worldwide, no approved vaccine or effective antiviral therapy is available. Although RSV infections do evoke humoral and cellular immune responses required to clear infection, these responses do not provide strong protection against a subsequent infection with RSV. Early during infection, viral replication is sensed by the host’s innate immune system, which leads to the production of type I and III interferons (IFNs), which is followed by the induction of an array of genes that code for antiviral proteins. In addition, IFN will recruit and activate innate leukocytes, including antiviral monocytes and natural killer (NK) cells, and stimulate the adaptive immune response [4]. RSV has evolved a marvelous set of mechanisms to subvert this canonical antiviral response of the mammalian host, most notably by virtue of its nonstructural (NS) 1 and NS2 proteins.

## Induction of type I and III IFN upon RSV infection

**Fig 1:**
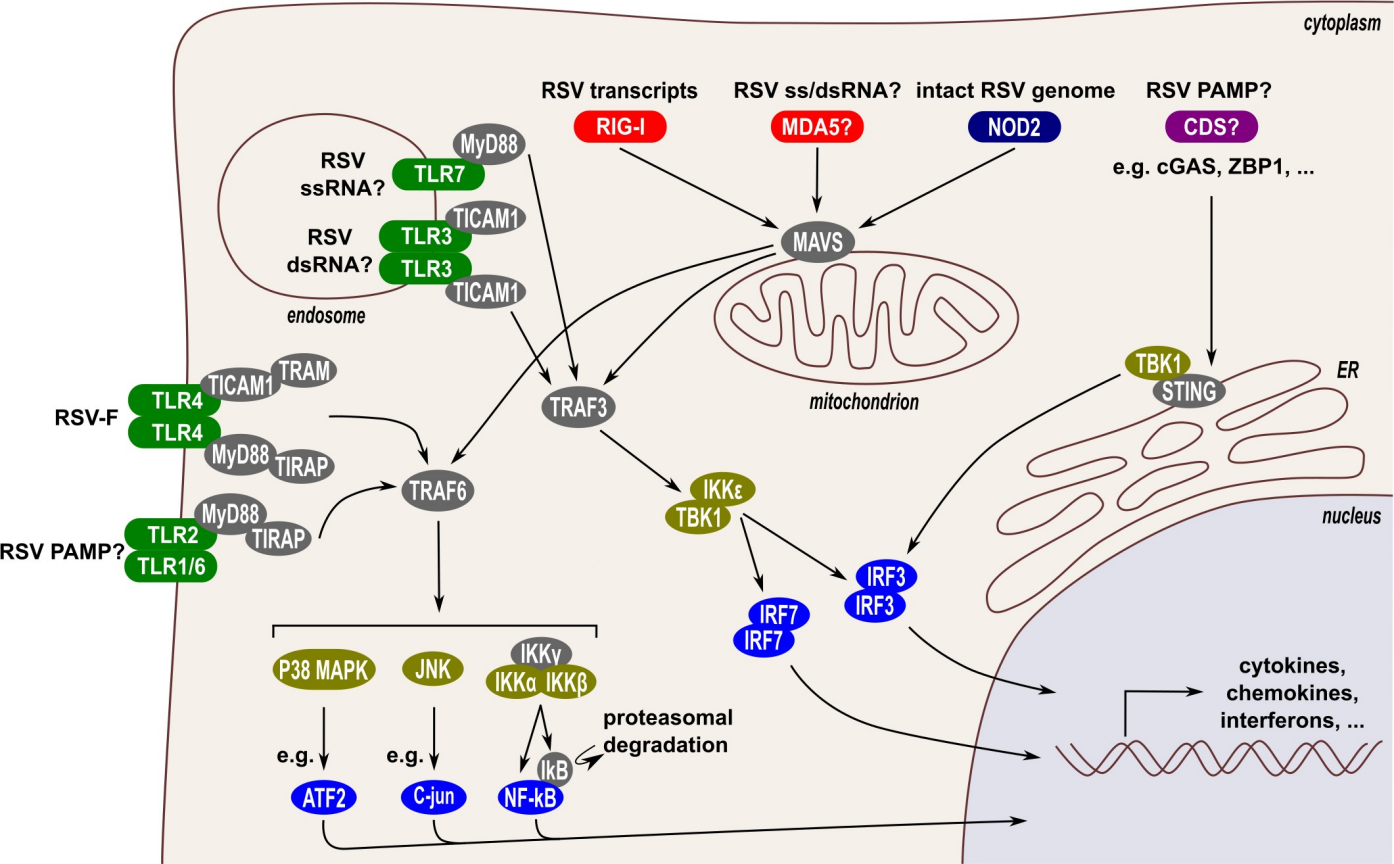
RSV-induced innate immune responses. Different families of PRRs can induce innate immune responses after activation by RSV-derived PAMPs. Firstly, TLR-2, -3, -4, -6, and -7 (marked in green) are involved in the production of cytokines and chemokines upon RSV infection. Secondly, RIG-I and possibly MDA5 (RLRs, marked in red), are important in the induction of type I IFNs. Thirdly, NOD2 (marked in dark blue) also induces type I IFNs upon RSV infection. Currently, there is no evidence for a role of the CDSs (marked in purple), which signal through the ER-associated STING protein, as PRRs during RSV infection. Activation of the RLRs or NOD2 induces their association with the mitochondrial-associated MAVS, which recruits the adaptor proteins TRAF3 or TRAF6. Via the TRAF3 adaptor, the kinases IKKε and TBK1 are subsequently activated, which phosphorylate and activate the transcription factors IRF3 and IRF7. Via the TRAF6 adaptor, 3 kinases are activated, i.e., the IKK kinase complex, JNK, and p38 MAPK, which phosphorylate and activate multiple transcription factors such as NF-κB, C-Jun, and ATF2, respectively. Activation of TLRs leads to the recruitment of adaptor proteins, e.g., MyD88, TICAM1, TIRAP, and TRAM. These adaptors can signal via TRAF3 or TRAF6. The transcription factors activated by PRR signaling ultimately induce expression of cytokines, chemokines, and IFNs. Above each PRR, the confirmed or likely RSV-derived PAMP is depicted. ATF2, activating transcription factor 2; CDS, cytoplasmic DNA sensor; ER, endoplasmic reticulum; IFN, interferon; IKK, inhibitor of nuclear factor kappa-B kinase; IKKε, inhibitor of nuclear factor kappa-B kinase subunit epsilon; IRF3, interferon regulatory factor 3; IRF7, interferon regulatory factor 7; JNK, c-Jun N-terminal kinase; MAPK, mitogen-activated protein kinase; MAVS, mitochondrial antiviral-signaling protein; MDA5, melanoma differentiation-associated protein 5; MyD88, myeloid differentiation primary response protein MyD88; NF-kB, nuclear factor-kappa B; NOD2, nucleotide-binding oligomerization domain-containing protein 2; PAMP, pathogen-associated molecular pattern; PRR, pattern recognition receptor; RIG, retinoic-acid-inducible gene-I; RLR, RIG-I-like receptor; RSV, respiratory syncytial virus; STING, stimulator of interferon protein; TBK1, tank binding kinase 1; TICAM1, toll/interleukin-1 receptor domain-containing adapter molecule 1; TIRAP, toll/interleukin-1 receptor domain-containing adapter protein; TLR, toll-like receptor; TRAF3, tumor necrosis factor receptor-associated factor 3; TRAF6, tumor necrosis factor receptor-associated factor 6; TRAM, toll-like receptor adaptor molecule.

The innate immune system is an important early line of defense against pathogens. This system comprises pattern recognition receptors (PRRs) that can recognize pathogen-associated molecular patterns (PAMPs). Activated PRRs can induce the expression of cytokines, e.g., type I and III IFNs, which mount an antiviral state in an autocrine and paracrine fashion. Upon RSV infection, airway epithelial cells, macrophages, and dendritic cells (DCs) are the main inducers of innate immune responses. Several Toll-like receptors (TLRs) have been identified that can act as PRRs for RSV-derived PAMPs (Fig 1). TLR2, -3, and -6, for example, have been implicated in the induction of cytokines and chemokines upon RSV infection [5, 6]. A role for TLR4, which is well known to respond to lipopolysaccharide, as a PRR for RSV is currently debated. Although some groups reported an impaired innate immune response in TLR4-deficient mice [7–9], Ehl and colleagues could not confirm a role for a TLR4-mediated immune response upon RSV infection [10]. In addition, TLR7 might even play a role in tempering immune responses upon RSV infection [11]. Moreover, type I IFN production by macrophages and DCs from wild-type and TLR1, -2, -4, -6, and -7 knockout mice is very similar following RSV infection [5, 11]. Finally, alveolar macrophages are the primary source of type I IFN in RSV-infected mice, and TLRs do not play a crucial role in this response [4, 12].

In contrast, retinoic-acid-inducible gene-I (RIG-I)-like receptors (RLRs) are important for the induction of type I and possibly type III IFNs upon recognition of RSV (Fig 1). Type I IFN expression is strongly hampered in the absence of mitochondrial antiviral-signaling protein (MAVS), the adaptor protein for RIG-I and melanoma differentiation–associated protein 5 (MDA5) [12, 13]. Early on, gene expression ablation studies revealed that RIG-I is the most important RLR to detect RSV, which is supported by the observation that RSV mRNA could be coimmunoprecipitated with RIG-I but not with MDA5 [14–17]. Later, however, both RIG-I and MDA5 were found to colocalize with RSV genomic RNA and the N protein [18]. Interestingly, whereas RIG-I partially localizes to RSV-induced inclusion bodies, MDA5 and MAVS were found nearly exclusively in these inclusion bodies, thereby dramatically blunting IFN-β induction.

A third class of PRRs, the nucleotide-binding oligomerization domain-like receptors (NLRs), is also important for the recognition of RSV. Nucleotide-binding oligomerization domain-containing protein 2 (NOD2) can be activated by intact genomic single-stranded RNA (ssRNA) and is involved in the induction of IFN-β in mice in a MAVS-dependent way (Fig 1) [19].

Cytoplasmic DNA sensors (CDSs), such as Z-DNA binding protein 1 (ZBP1) and cyclic GMP-AMP synthase (cGAS), are well known to induce innate immune responses upon recognition of pathogen-derived double-stranded DNA (dsDNA) or even RNA [20–22]. A contribution of CDSs in innate sensing of RSV replication has not yet been reported.

## How RSV NS protein 1 and 2 overcome innate responses

In contrast to other respiratory viruses, i.e., influenza virus and human respirovirus 1 (formerly named human parainfluenza virus 1), nasal washes from RSV-infected infants hardly contain IFN-α and -β [23–26]. Apparently, this virus has evolved ways to outsmart the canonical mammalian antiviral response. Two unique viral proteins, NS1 and NS2, are responsible for the suppression of IFN induction and signaling. Human and bovine RSV strains that lack NS1 and/or NS2 have been explored as live-attenuated vaccine candidates. Such viruses are strongly attenuated in in vivo RSV infection models (cotton rats, calves, and chimpanzees) as well as in human but, at least in calves and chimpanzees, retain their ability to induce antibody responses [27–36]. Positioned 3′ proximal on the negative-stranded RNA genome, NS1 and NS2 are the most abundantly transcribed viral genes. Recently, the crystal structure of NS1 was determined, revealing that this protein is composed of a β-sandwich flanked by 3 α-helices [37]. Interestingly, the 3D structure of NS1 is very similar to the N-terminal domain of RSV M despite the complete absence of any primary sequence homology.

Both NS1 and NS2 strongly reduce the induction of type I and III IFNs upon RSV infection [32, 37–41]. Infection with recombinant viruses lacking NS1 or NS2, separately or combined, suggests that these proteins function individually and cooperatively to suppress IFN induction. They do so by targeting multiple proteins of the signaling cascade that starts with the recognition of PAMPs by PRRs and ends with the induction of IFN gene expression by several transcription factors.

### NS1 and NS2 interfere with RIG-I

**Fig 2:**
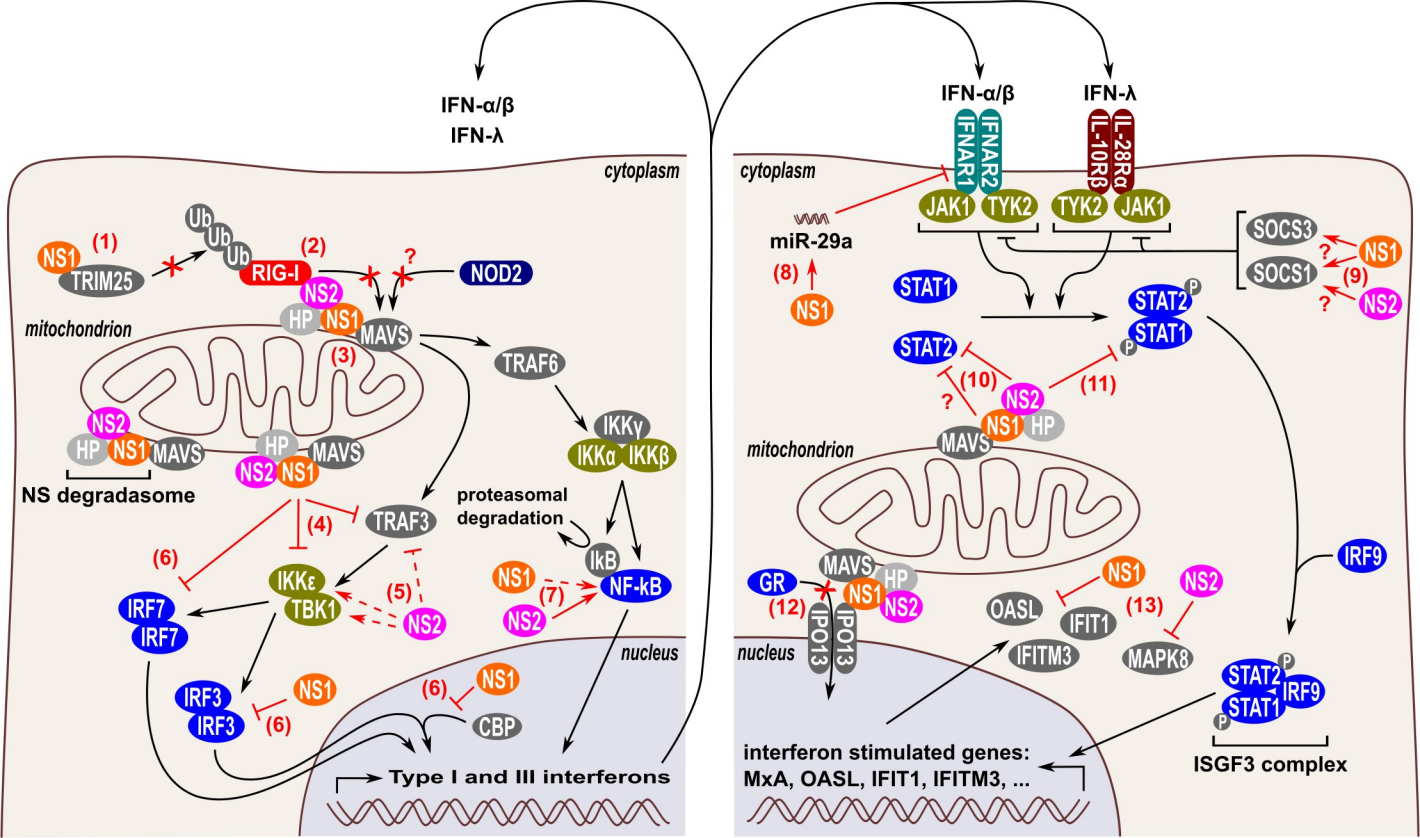
RSV NS1 and NS2 effector functions that can suppress IFN responses. Type I and III IFN responses are inhibited by NS1 and NS2 at multiple levels, both during the induction of type I and III IFNs (left panel) and during IFN-induced signaling (right panel). NS1 and NS2 can form a so-called “NS degradasome” complex that is stabilized by mitochondria via MAVS. The NSD complex is thought to contain HPs, including the proteasome α2 subunit and other as yet unidentified proteins. NS1 and NS2 prevent the interaction of RIG-I with MAVS in different ways. NS1 binds to the PRY-SPRY domain of TRIM25, which is responsible for the interaction of TRIM25 with RIG-I. As such, NS1 prevents the TRIM25-mediated K63-linked polyubiquitination of RIG-I, which is necessary for the subsequent interaction of RIG-I with MAVS (1). Moreover, NS2 directly interacts with RIG-I (2) and NS1 interacts with MAVS (3) to suppress binding of RIG-I to MAVS. Whether the interaction of NS1 with MAVS also prevents the interaction of NOD2 with MAVS is currently unclear. NS1 reduces protein expression of TRAF3 and IKKε (4), whereas NS2 modestly reduces TRAF3 and induces IKKε and TBK1 (5). NS1 subsequently inhibits IRF3 and IRF7 by different proposed mechanisms (6). NS1 reduces IRF3 and IRF7 protein expression and prevents the interaction between IRF3 and CBP, thereby lowering the binding of the IRF3-CBP complex to the IFN-β promoter. NS2, and to a lesser extent NS1, enhances the activation and nuclear translocation of NF-κB (7). These NS1/NS2 effector functions (1–7) synergistically reduce the production of type I and III IFNs. Furthermore, NS1 and NS2 also suppress type I and III IFN receptor-mediated signal transduction. NS1 induces miR-29a expression, which targets the mRNA coding for IFNAR1, one of the 2 subunits of the type I IFN receptor (8). NS1 and NS2 may induce expression of SOCS proteins (SOCS1 and 3), which negatively regulate the tyrosine kinases JAK1 and TYK2, which are important to transmit signaling from the type I and III IFN receptors (9). NS2 inhibits JAK1/TYK2-mediated activation of STAT1/2 by reducing STAT2 protein levels (10) and by reducing STAT1 phosphorylation (11). Some groups, however, reported that STAT2 expression can also be reduced by NS1 (see text) (10). NS1 and NS2 counteract the anti-inflammatory activity of the GR, although the exact mechanism is debated. In one model, NS1 interacts with the nuclear translocator IPO13, which competes with GR for its nuclear translocation (12). Recent evidence suggests that the NS proteins may also counteract antiviral effector functions of ISGs, e.g., NS1 degrades the OASL, IFIT1, and IFITM3 proteins, whereas NS2 degrades MAPK8 (13). Full and dashed lines indicate robust and moderate inhibitory or stimulatory effector functions, respectively. CBP, CREB binding protein; GR, glucocorticoid receptor; HP, host protein; IFIT, interferon-induced protein with tetratricopeptide repeats; IFITM, interferon-induced transmembrane protein; IFN, interferon; IFNAR, interferon alpha/beta receptor; IKKε, inhibitor of nuclear factor-kappa B kinase subunit epsilon; IPO13, importin-13; IRF3, interferon regulatory factor 3; ISG, IFN-stimulated gene; JAK, janus kinase; MAPK8, mitogen-activated protein kinase 8; MAVS, mitochondrial antiviral-signaling protein; NS, nonstructural; OASL, 2′-5′-oligoadenylate synthase-like protein; RIG-I, retinoic-acid-inducible gene-I; RLR, RIG-I-like receptor; RSV, respiratory syncytial virus; SOCS, suppressor of cytokine signaling; STAT, signal transducer and activator of transcription; TBK1, tank binding kinase 1; TRIM25, tripartite motif–containing protein; TYK, tyrosine kinase 25.

A widespread strategy of viruses to dampen innate immune responses is to counteract one of the early signaling steps in RLR-mediated IFN induction, i.e., the interaction of RIG-I or MDA5 with MAVS [42–45]. Likewise, RSV prevents the interaction of RIG-I with its adaptor MAVS. In RSV-infected HEp-2 cells and A549 cells overexpressing NS1, NS1 was shown to interact with MAVS (Fig 2) [46]. By binding to MAVS, NS1 could dose-dependently prevent the interaction between RIG-I and MAVS in A549 cells. Furthermore, Ban and colleagues demonstrated that ectopically expressed NS1 in HEK293T cells interacts with the PRY-SPRY domain of E3 ubiquitin/IFN-stimulated gene 15 (ISG15) ligase tripartite motif–containing protein 25 (TRIM25). This domain is responsible for the interaction of TRIM25 with RIG-I [47]. NS1 binding to the PRY-SPRY domain suppresses K63-linked polyubiquitination of RIG-I by TRIM25, which is essential for its downstream interaction with MAVS (Fig 2). Proteins of 2 other respiratory viruses also target the PRY-SPRY domain of TRIM25, which highlights the evolutionary importance of TRIM25 in mammalian antiviral defense. By binding the PRY-SPRY domain of TRIM25, the NS1 protein of influenza A virus and the nucleocapsid protein of severe acute respiratory syndrome virus also counteract TRIM25-mediated RIG-I ubiquitination [48, 49]. NS2 can interact with the N-terminal domain of RIG-I, both upon overexpression in HEK293T cells and during RSV infection in A549 cells (Fig 2) [50]. As such, overexpressed NS2 disrupts the binding of RIG-I with MAVS; however, this has not yet been confirmed for endogenous NS2 expressed during an RSV infection. In addition to counteracting the interaction between RIG-I and MAVS, NS1 may also influence RIG-I expression, although the reported findings seem conflicting. RIG-I expression in A549 cells is strongly reduced in the presence of RSV NS1, either expressed separately or in the context of an RSV infection [51]. Boyapalle and colleagues, however, reported that RIG-I expression in A549 cells is reduced following infection with an NS1-deficient RSV [46]. This is surprising, because RIG-I is itself an ISG. Possibly, this discrepancy is caused by the different multiplicity of infection (MOI) used by these 2 groups (MOI 3 and 1, respectively). In contrast to RIG-I, MAVS appears to be resistant to NS1- and/or NS2-mediated down-regulation [51]. All together, these results highlight that the interaction of RIG-I with MAVS is suppressed by both NS1 and NS2 and that RIG-I expression itself might be reduced by NS1. Currently, it is not clear whether NS1 and/or NS2 can disturb the interaction of MDA5 or NOD2 with MAVS and whether they impact MDA5 or NOD2 expression levels.

### NS1 and NS2 interfere with signaling downstream of MAVS

Association of RLRs with MAVS leads to the recruitment of the adaptors tumor necrosis factor receptor-associated factor 3 (TRAF3) and -6 (TRAF6). Whereas TRAF3 activates the downstream serine/threonine kinases inhibitor of nuclear factor-kappa B kinase subunit epsilon (IKKε) and tank binding kinase 1 (TBK1), TRAF6 activates the downstream kinases IKK, c-Jun N-terminal kinase (JNK), and p38 mitogen-activated protein kinase (p38 MAPK) (Fig 1). The effect of NS1 and NS2 on TRAF3, IKKε, and TBK1 is currently inconclusive. Some groups reported that NS1, either overexpressed or expressed during an RSV infection in A549 cells, reduces the expression levels of both TRAF3 and IKKε (Fig 2) [51, 52]. Ren and colleagues, however, observed no difference in endogenous and recombinant TRAF3 and IKKε expression in A549 cells in the presence or absence of NS1 [39]. Possibly, the different strains (RSV long versus A2) or experimental timing used explain these opposing observations. Overexpression of NS2 in A549 cells modestly reduces and slightly enhances TRAF3 and IKKε expression levels, respectively (Fig 2) [51, 52]. These effects of NS2 were, however, not observed with an RSV strain that lacks NS1. Ling and colleagues also observed that overexpressed NS2 enhances overexpression of IKKε and TBK1 in HEK293T cells (Fig 2) [50]. To our knowledge, no data have been published on the possible effect of NS1 on the expression of TBK1. Coexpression of NS1 and NS2 in A549 cells reduces recombinant IKKε expression, suggesting that the inhibitory effect of NS1 is dominant over the enhancing effect of NS2 [52]. These results suggest that NS1 suppresses TRAF3 and IKKε expression, although more evidence is needed to confirm this hypothesis. Ectopic expression of NS2 slightly suppresses TRAF3 expression and enhances IKKε and TBK1 expression, although these effects need to be confirmed during an RSV infection.

Whether NS1 and/or NS2 affect the TRAF6 adaptor has not been investigated yet. Some evidence indicates that RSV may indeed affect TRAF6 expression. In different models, TRAF6 expression has been shown to be down-regulated by the microRNA (miRNA) miR-146a [53, 54]. Interestingly, Eilam-Frenkel and colleagues found that miR-146a expression was up-regulated in RSV-infected HEp-2 cells [55]. Future research may elucidate whether RSV can down-regulate TRAF6 expression via up-regulating miR-146a and whether this is mediated by NS1 and/or NS2.

### NS1 and NS2 interfere with the interferon regulatory factor and nuclear factor-kappa B transcription factors

Activated IKKε and TBK1 phosphorylate the transcription factors Interferon Regulatory Factor (IRF)-3 and IRF7, which induces conformational changes that allow the formation of homo- and heterodimers of IRF3 and -7 that translocate to the nucleus. Phosphorylation of inhibitor of kappa B (IκB), e.g., by the canonical IKKα/β/γ complex, initiates its degradation with subsequent release and nuclear translocation of nuclear factor-kappa B (NF-κB). The IRF and NF-κB transcription factors are essential for the induction of type I and III IFNs (Fig 1).

Several observations highlight that NS1 and NS2 can impair the activation and effector functions of IRF transcription factors. Initial work with recombinant RSV strains lacking one or both NS proteins in A549 cells highlighted that NS1 and NS2 prevent nuclear translocation of IRF3, especially late (>9 hours post infection) in the RSV replication cycle [40, 50, 56]. This might, in part, be explained by the ability of NS1 to directly reduce the recombinant expression of IRF3 and IRF7 (Fig 2) [51]. In addition, by preventing the formation of the IRF3/CREB binding protein (CBP) complex in the nucleus, NS1 may also inhibit IRF3-dependent gene expression downstream of the nuclear translocation of IRF3 (Fig 2) [37, 39]. It is important to note that this NS1 effector function has only been demonstrated upon overexpression in HEK293T cells, so additional confirmation in an RSV infection is necessary.

In A549 and Vero cells, NF-κB activation and nuclear translocation occur early after RSV infection and are clearly enhanced by NS2 and, to some extent, by NS1 (Fig 2) [56, 57]. In addition to the canonical IκB phosphorylation and subsequent degradation, RSV-induced NF-κB activation involves p65Ser536 phosphorylation via the RIG-I/MAVS/TRAF6/IKKβ signaling pathway [16]. Although NF-κB contributes to the induction of type I and III IFNs, NF-κB also strongly induces the expression of anti-apoptotic genes. In the context of an RSV infection, the induction of an anti-apoptotic cellular environment by NF-κB likely dominates the contribution of NF-κB over the induction of IFN, which is primarily controlled by IRF3/7 activation.

Taken together, NS1 and NS2 suppress the induction of type I and III IFNs by targeting multiple proteins of the signaling cascade that leads to type I or III gene activation, i.e., RIG-I, MAVS, TRAF3, IKKε, TBK1, IRF3, and IRF7. Although some of these effector functions have been confirmed in RSV-infected cells, others have so far only been identified with overexpression of NS1 or NS2 alone. So, future research is needed to elucidate the biological relevance of these recombinant responses, particularly because NS2 can relocate NS1 to the mitochondria [58].

### NS1 and NS2 suppress type I and III IFN receptor–mediated signal transduction

Binding of type I and III IFNs to their heterodimeric receptor complex induces a Janus kinase (JAK)- signal transducer and activator of transcription (STAT) signaling cascade that induces an antiviral state by expression of ISGs. Compared with wild-type RSV, RSV strains that lack NS1 and/or NS2 are more sensitive to type I IFN treatment [30, 51]. Thus, RSV NS proteins also have effector functions downstream of the induction of IFN synthesis to suppress IFN-induced antiviral responses.

In RSV-infected A549 cells, NS1 induces the expression of the miRNA miR-29a, which targets the mRNA coding for interferon alpha/beta receptor 1 (IFNAR1) by an unknown mechanism (Fig 2) [59]. As a result, the IFNAR1 protein levels are reduced, which decreases responsiveness to type I IFNs of RSV-infected cells and thus favors RSV replication. The induction of an antiviral state by type I and type III IFNs requires the phosphorylation of STAT1 and STAT2 proteins in the proximity of the type I and III receptors.

Two tyrosine kinases, JAK1 and tyrosine kinase 2 (Tyk2), are responsible for the phosphorylation and activation of STAT proteins after activation of the type I and III IFN receptor (Fig 2). For now, there is no evidence that NS1 or NS2 may alter the levels and activation status of these 2 kinases. Several groups reported that the phosphorylation and total protein levels of Tyk2 are not altered by the expression of NS1 and/or NS2 [60–62]. Whether this also accounts for JAK1 is currently not clear. Infection of airway epithelial cell lines with human metapneumovirus, a respiratory virus closely related to RSV, does reduce both JAK1 and Tyk2 protein levels [63].

Additionally, JAK kinase activity is regulated by a negative feedback loop that consists of the suppressor of cytokine signaling (SOCS) family, with SOCS1 and 3 being the strongest inhibitors of JAK kinases. It has been reported that NS1 and/or NS2 may regulate the expression of SOCS1 and/or SOCS3, thereby enhancing RSV replication [64–67]. Heterodimers of phosphorylated STAT1 and 2 transcription factors are important for the induction of ISGs. Consequently, numerous viral proteins counteract STAT1 and 2 proteins by using different strategies, e.g., by preventing STAT1/2 heterodimer formation (Nipah and Hendra virus nucleoprotein), by suppressing STAT1/2 nuclear translocation (Ebolavirus VP24), or by degrading STAT1 and/or 2 (Paramyxovirus V protein) [68–70]. Both STAT1 and 2 are targets for RSV. In human tracheobronchial epithelial cells and A549 cells, infection with RSV slightly increases total STAT1 protein levels, likely because STAT proteins are themselves ISG products [60–62, 71, 72]. STAT1 phosphorylation induced by exogenous type I IFN, however, is clearly reduced by NS2 but not by NS1 (Fig 2) [60–62, 73]. Whether NS2 also reduces STAT1 phosphorylation during an RSV infection has not been reported yet. In contrast to STAT1, both phosphorylated and nonphosphorylated STAT2 protein levels are clearly reduced by RSV infection [60, 62, 73]. Whether NS1, NS2, or both reduce STAT2 levels is currently debated. Based on overexpression experiments and infections with RSV strains lacking NS1 and/or NS2, several groups reported that NS2, but not NS1, reduces STAT2 levels in vitro (Fig 2) [51, 52, 61, 62]. Others, however, also observed reduced STAT2 levels after overexpression of NS1 (Fig 2) [66, 73]. Lo and colleagues reported that recombinant coexpression of NS1 and NS2 reduces STAT2 levels stronger than NS2 alone, although expression of NS1 alone did not affect STAT2 levels [62]. In addition to reducing total STAT2 levels, RSV also seems to suppress nuclear translocation of the residual STAT2 proteins, an effect that depends on NS1 and/or NS2 [62]. So far, the possible impact of RSV NS proteins on STAT1 and 2 expression levels in vivo has not yet been demonstrated. Because mouse STAT2 appears resistant to NS2, primary human airway epithelial cell cultures or experiments in nonhuman primates may be required to confirm that NS2-mediated STAT2 reduction also occurs in vivo [62].

### NS1 and NS2 interfere with ISG products

Evidence is rising that the NS proteins also suppress the antiviral activity of at least some ISG products. Cells that express myxovirus resistance protein A (MxA) or cells pretreated with type I IFN only modestly limit RSV replication [74]. Moreover, ectopic expression of NS1 and NS2 in HEK293-derived cells has been shown to degrade certain ISG products, i.e., 2′-5′ oligoadenylate synthetase–like protein (OASL), interferon-induced protein with tetratricopeptide repeats 1 (IFIT1), interferon-induced transmembrane protein 3 (IFITM3), and MAPK8, in a selective manner (Fig 2) [75, 76]. An important remark, however, is that these effector functions have not yet been validated during an RSV infection. As stated by Ribaudo and Barik, a comprehensive screen of all known ISGs will likely unravel additional substrates of NS1 and/or NS2 [76].

### Other effector functions of RSV NS1 and NS2

#### NS1 and NS2 affect the induction of apoptosis and cell shedding

NS1 and NS2 individually and cooperatively delay apoptosis in RSV-infected cells, with NS2 being stronger than NS1 in doing so [57]. The early expression of NS1 and NS2 after infection activates the anti-apoptotic 3-phophoinositide-dependent protein kinase (PDK)-RAC serine/threonine-protein kinase (AKT)-glycogen synthase kinase (GSK) pathway [56, 57, 77]. Later in infection (>24 hours), activation of this pathway drops, and the incidence of apoptosis increases [57]. By delaying apoptosis, NS1 and NS2 may facilitate prolonged RSV replication with increased viral yields.

A typical hallmark of severe disease following RSV infection is the obstruction of the smaller airways by plugs consisting of infiltrating immune cells, mucus, and shed infected epithelial cells. In primary human airway epithelial cells and in an in vivo hamster model, it has been shown by experiments with a set of elegant recombinant virus constructs that RSV NS2 is necessary and sufficient to induce shedding of infected epithelial cells [78]. Interestingly, cell death, associated with nuclear changes that are indicative of apoptosis, of infected epithelial cells only occurred after these cells were detached from the epithelial layer. Moreover, shedding of infected epithelial cells coincided with reducing viral titers.

All together, these results suggest that expression of NS2 early after infection delays apoptosis and induces changes in cell morphology that ultimately result in shedding of infected cells in the airway lumen. These shed and detached infected epithelial cells die. As shedding (and ultimately clearance by mucociliary transport) of infected epithelial cells reduces RSV viral titers, it seems that the bulk of viral spread precedes cell shedding. Possibly, (early) changes in cell morphology that ultimately lead to shedding of infected epithelial cells may facilitate RSV production and spreading. As such, NS2 plays a pivotal role in the production and spreading of RSV virions.

#### NS1 and NS2 counteract the anti-inflammatory activity of the glucocorticoid receptor

Although RSV-induced bronchiolitis is characterized by a (severe) inflammatory response, the use of anti-inflammatory glucocorticoids has shown no clinical benefits against (severe) disease [79–81]. Based on experiments with different transformed and primary cell cultures as well as mice, several groups concluded that RSV can inhibit the anti-inflammatory activity of glucocorticoids via the glucocorticoid receptor (GR) [82–88]. The exact mechanism that accounts for this inhibition, however, is debated. One group demonstrated that RSV blocks glucocorticoid-mediated GR activation in A549, BEAS-2B, and primary human small airway epithelial cells but not in the monocytic THP-1 cell line, suggesting that the effect is restricted to RSV-infected epithelial cells [83, 85]. Further mechanistic analysis in A549 cells suggested that NS1 and NS2 reduce the binding of the GR to GR-responsive promoters, without affecting GR total protein levels and nuclear translocation [83, 84, 86]. Interestingly, knockdown of MAVS in A549 cells lowered dexamethasone-induced transforming growth factor β (TGF-β)-stimulated clone 22 (TSC22) domain family member 3 (also known as glucocorticoid-induced leucine zipper protein [GILZ]) mRNA expression to a level similar to that of an RSV infection [86]. These results suggest that MAVS plays a role in glucocorticoid-induced GR activation in A549 cells and that NS1 and NS2 may indirectly suppress GR activation through inhibition of MAVS (see “NS1 and NS2 interfere with RIG-I”).

Xia and colleagues used BEAS-2B and primary differentiated human bronchial epithelial cells grown at an air–liquid interface to demonstrate that RSV suppresses glucocorticoid-induced GR activation by a mechanism that involves up-regulation of TGF-β expression [87]. A model was proposed in which viral infection is sensed by TLR3, which induces TGF-β that subsequently activates the type I TGF-β receptor activin receptor-like kinase 5 (ALK5). ALK5 activation subsequently counteracts glucocorticoid activity through an unknown mechanism. In accordance with the aforementioned study, no difference in total GRα protein levels or nuclear translocation were observed upon RSV infection. Interestingly, blocking ALK5 with the selective inhibitor SB431542 and reducing TGF-β activity by tranilast (a compound used to manage a wide variety of diseases, including inflammatory diseases) could subvert the RSV-mediated suppression of glucocorticoid activity. Although Xia and colleagues did not investigate the role of NS1 or NS2, NS1 may contribute to RSV-induced TGF-β expression by up-regulating the transcription factor Kruppel-like factor 6 (discussed in the next paragraph) [89].

Another study concluded that RSV NS1 prevents GR nuclear translocation [88]. This finding was based on experiments with A549 cells, mouse lung tissue, but also analysis of nasopharyngeal aspirates from RSV-infected infants. Moreover, expression of GRα and importin-13, which is important for GR nuclear entry, was reduced at the mRNA and protein level in mouse lungs, whereas GRβ and IPO13 expression at the mRNA level were reduced in the nasopharyngeal aspirates upon RSV infection. Mechanistically, NS1 was found to directly interact with IPO13 and can thus compete with GR for IPO13 binding.

The capacity of RSV to suppress the anti-inflammatory effect of glucocorticoids through NS1 and NS2 are in line with the ineffectiveness of glucocorticoid treatments off severely ill RSV patients.

#### NS1 and NS2 interfere with the induction of miRNAs

In addition to miR-29a, NS proteins also regulate the expression of the miRNAs miR-24, let-7i, and miR-30b [59, 89, 90]. In RSV-infected A549 cells, NS1 suppresses miR-24 expression by up-regulating the transcription factor Kruppel-like factor 6, which drives the expression of TGF-β [89]. Remarkably, inhibition of miR-24 was shown to actually repress RSV replication in A549 cells [91]. This apparent discrepancy could be explained by the difference in time points after infection that were analyzed (1 day versus 3 days post infection). Possibly, NS1 suppresses the RSV-induced up-regulation of miR-24 within 24 hours post infection, whereas later on, miR-24 expression may be up-regulated to enhance viral replication. Investigating miR-24 expression levels beyond 24 hours post infection and the impact of NS1 on these levels may result in a better understanding of the role of miR-24 in RSV replication. In normal human bronchial epithelial cells, Let-7i and miR-30b expression is up-regulated during an RSV infection by a type I IFN and NF-κB-dependent mechanism, respectively, and further increased in the absence of NS1 or NS2 [90]. This can be expected for let-7i, as the NS proteins suppress the type I IFN response. Moreover, the miR-30b promoter can be activated by the NF-κB family member p65, which is activated during an RSV infection through a RIG-I/MAVS/TRAF6/IKKβ signaling pathway [16, 92]. By counteracting the interaction between RIG-I and MAVS (see “NS1 and NS2 interfere with RIG-I”), the NS proteins may dampen this pathway, leading to reduced activation of p65 and subsequent expression of miR-30b. To our knowledge, the effect of NS1 and/or NS2 on the RSV-induced activation of p65 has not been investigated yet. This could readily be tested by comparing total levels of activated p65 between cells infected with wild-type RSV and NS deletion strains. Whether the NS protein-mediated suppression of let7i and miR-30b favors or counteracts RSV replication is currently unclear.

#### NS1 and NS2 interfere with the adaptive immune response

The NS proteins also play a role in the development of adaptive immune responses during RSV infection, partly as a consequence of their capacity to suppress type I and III IFN levels. In addition to airway epithelial cells, RSV can infect DCs and activate PRRs by PAMPs [93]. Munir and colleagues investigated the activation status of isolated human monocyte–derived myeloid DCs upon RSV infection by quantifying several maturation markers, including cluster of differentiation (CD)38, 54, 80, 83, and 86 [41]. Infection with wild-type and NS-deficient RSV strains highlighted that NS1, and to a lesser extent NS2, suppress the maturation of human DCs. By using an IFNAR2-blocking antibody, this suppression was shown to partially depend on the capacity of the NS proteins to counteract the production of type I IFNs. Moreover, in vivo pulmonary conventional DC activation, as measured by the up-regulation of CD86 and CD80, was strongly hampered in RSV-infected mice that are deficient for MAVS, an essential signaling protein for RSV-induced type I IFN production [12]. Taken together, these results highlight that type I IFNs play a role in RSV-induced DC maturation. As such, the pleiotropic effector functions of NS1 and NS2 to counteract the production of type I IFNs likely account for the reduced DC maturation by NS1 and NS2.

In a follow-up report, Munir and colleagues investigated the consequence of reduced DC maturation by NS1 and NS2 on subsequent activation of T-cell responses by co-cultivation of RSV-infected human monocyte-derived DCs and autologous T cells [94]. Deletion of NS1 was found to promote the proliferation and activation of CD103^+^ CD8^+^ T cells and T-helper 17 cells, 2 cell populations that can counteract RSV, and to suppress the activation of IL-4-producing CD4^+^ T cells. Remarkably, none of these effects on T cells appeared to depend on type I IFN. Possibly, IL-12β and IL-23α play a role as these cytokines were suppressed in DCs by NS1 in a type I IFN–independent manner. In contrast, a study by Kotelkin and colleagues in mice identified that NS2, and not NS1, suppresses CD8^+^ T-cell responses in a type I IFN–dependent manner [95]. Remarkably, although RSV-induced activation of DCs in MAVS^-/-^ and MAVS^-/-^myeloid differentiation primary response protein MyD88 (MyD88)^-/-^ mice was strongly impaired, these mice could still mount comparable CD8^+^ T-cell responses as wild-type mice [12]. This may in part be explained by the approximately 100-fold higher viral loads in MAVS^-/-^ mice compared with wild-type mice. In addition, a small residual population of CD86-positive pulmonary DCs was still present in these mice that could migrate to the draining mediastinal lymph node to activate naive CD8^+^ T cells. As MAVS^-/-^MyD88^-/-^ mice are still functional for toll/interleukin-1 receptor domain-containing adapter molecule 1 (TICAM1), the adaptor of TLR3, these DCs may have matured after the activation of TLR3 by RSV-derived double-stranded RNA (dsRNA) [96].

Live-attenuated RSV vaccines with a targeted deletion in NS2 have been tested for use in infants [35]. Whereas a strain with only NS2 deleted was insufficiently attenuated, 2 other NS2 deletion strains with additional mutations were found to be over-attenuated, highlighting the importance of a right balance between attenuation and immunogenicity. Currently, 2 phase I clinical trials with other NS2 deletion strains (ClinicalTrials.gov Identifiers: NCT03422237 and NCT03387137) and one with an NS1 deletion (ClinicalTrials.gov Identifier: NCT03596801) are ongoing. The primary endpoints of these ongoing phase I trials with the live-attenuated RSV strains that lack either NS1 or NS2 are measures for safety, vaccine virus infectivity, and the induction of RSV-neutralizing titers. To our knowledge, cellular immune responses after immunization with these live-attenuated RSV vaccines have not been investigated.

### Mechanism(s) of effector functions

Taking into account the small size of NS1 and NS2, it is remarkable how many host functions are affected by these RSV proteins. Goswami and colleagues hypothesized that NS1 and NS2 form a large (300 to 750 kDa) degradative complex, which they called the NS degradasome (NSD) [51]. In A549 cells, this complex could selectively degrade particular innate immune signaling proteins of which RIG-I was also confirmed as a substrate of NSD complexes isolated from RSV-infected cells. In this respect, it would be interesting to assess whether other reported NS target proteins are also substrates of the proposed NSD complexes in RSV-infected cells. The NSD complex appears to be metastable, and its activity is enhanced by mitochondria via MAVS, possibly through stabilizing the NSD complex [51]. Interestingly, in MAVS-deficient cells stimulated with IFN-α, wild-type RSV is almost equally attenuated as RSV lacking NS1 and NS2, confirming the importance of MAVS for NS protein-mediated suppression of type I IFN responses. In line with these results, NS2 mainly localizes to mitochondria and seems to recruit NS1 towards the mitochondria [58]. Furthermore, NS target proteins, but not other innate proteins, relocate from the cytoplasm to the mitochondria upon expression of NS1 and NS2 [51]. All together, these results support the hypothesis of the formation of a mitochondrial-associated NSD complex to selectively degrade innate immune proteins in RSV-infected cells.

Although the exact composition of the NSD complex is still unresolved, the presence of the α2 subunit from the 20S core proteasome sparked the idea that the NSD complex might act in a similar way as the host 26S proteasome [51]. In line with this hypothesis, NS protein–induced STAT2 and OASL degradation is (partially) blocked by the proteasome inhibitors MG132 and lactacystin [51, 60, 61, 73, 75]. In contrast, NS protein–induced TRAF3 and IKKε degradation appears insensitive to MG132 [52]. Proteasome-like activity of the NSD complex is further supported by the identification of NS1 and NS2 as potential inducers of host protein ubiquitination [73, 97]. Whereas NS1 is a putative Elongin B/C-Cullin 2/5-SOCS box-type E3 ubiquitin ligase, NS2 does not contain an Elongin C binding consensus sequence [73, 97, 98]. Interestingly, an RSV strain with 3 mutated NS2 residues that appear essential for ubiquitination activity and NS2-mediated STAT2 degradation is nearly equally attenuated as an NS2 deletion strain [97]. These results suggest that NS2 and NS1 may induce ubiquitination of host proteins, which are then degraded by the NSD complex. It is currently not known, however, if NS1 and/or NS2 selectively mark innate immune proteins for degradation, which would explain the selective degradative activity of the NSD complex. Recently, the crystal structure of NS1 was determined and revealed a remarkable structural homology with the N-terminal domain of the matrix protein of RSV [37]. One clear difference between NS1 and M is the presence of an α-helix (called α3) at the C-terminus of NS1. A truncated NS1 lacking this helix or mutation of 3 residues that are important for the interaction of helix α3 with the rest of NS1 partially abrogates NS1-mediated suppression of IFN-β induction and attenuates recombinant RSV strains. These results confirm that helix α3 is important for at least some NS1-mediated effector functions. Unravelling the crystal structure of NS2 and the composition of the NSD complex would greatly enhance our understanding of the remarkable diverse effector functions of NS1 and NS2. Moreover, NS1 and/or NS2 may be attractive targets for antiviral therapy. Intranasal administration of nanoparticles carrying a plasmid encoding an NS1-targeting small interfering RNA (siRNA) in mice can reduce lung viral titers, airway hyperresponsiveness, and pulmonary inflammation, both in a prophylactic as well as therapeutic setting [99]. A recent small compound high-throughput screen revealed 4 candidate inhibitors of NS2, highlighting that NS2 could be targeted by a small compound drug [100].

## General conclusion

In humans, RSV infections induce remarkably low levels of IFN-α and -β compared with other respiratory viruses. This is largely the consequence of 2 unique viral proteins, NS1 and NS2, that strongly suppress the induction and signaling of IFN. In recent years, evidence is rising that, in addition, antiviral activities of ISGs may be hampered by the NS proteins. NS1 and NS2 exert additional functions, such as delaying cell apoptosis, and loss of NS1 is associated with a stronger adaptive immune response and reduced in vivo viral replication. NS1 and NS2 are thought to form a so-called “NS degradasome” complex, which may function as a proteasome-like complex that selectively degrades a plethora of innate immune proteins. Further insight in the composition of the NSD and the resolution of the structure of NS2 will likely help to explain the remarkable diverse effector functions of NS1 and NS2. Some effector functions, however, have so far only been identified in artificial cell systems and should be interpreted carefully. Large-scale protein–protein interaction screens, preferentially performed using multiple complementary protein–protein interaction techniques in the context of an RSV infection, will generate a more comprehensive list of host proteins that interact with NS1 and/or NS2. Such new knowledge, combined with co-crystal structure analysis of RSV NS1 and NS2 in complex with host protein factors, will be instrumental to design antiviral drugs that impact on the RSV–host interface and thereby complement directly acting antivirals.
